# *Gyrodactylus triglopsi* n. sp. (Monogenea: Gyrodactylidae) from the Gills of *Triglops nybelini* Jensen, 1944 (Teleostei: Cottidae) in the Barents Sea

**DOI:** 10.2478/s11686-020-00208-z

**Published:** 2020-04-28

**Authors:** Haakon Hansen, Anja Helene Alvestad, Ken MacKenzie, Mari Darrud, Egil Karlsbakk, Willy Hemmingsen, Per Arneberg

**Affiliations:** 1 Norwegian Veterinary Institute, P.O. Box 750 Sentrum, 0106 Oslo, Norway; 2 Institute of Marine Research, Nordnes, P. O. Box 1870, 5817 Bergen, Norway; 3 School of Biological Sciences (Zoology), University of Aberdeen, Tillydrone Avenue, Aberdeen, AB24 2TZ Scotland UK; 4 Institute of Marine Research, Fram Centre, P.O. Box 6606, 9296 Langnes, Norway; 5 Department of Biology, University of Bergen, P.O. Box 7803, 5020 Bergen, Norway; 6 Department of Arctic and Marine Biology, Faculty of Biosciences, Fisheries and Economics, University of Tromsø, 9037 Tromsø, Norway

**Keywords:** Gyrodactylidae, Cottidae, Bigeye sculpin, Barents sea

## Abstract

**Introduction:**

Monogeneans of the genus *Gyrodactylus* were found on the gills of specimens of the bigeye sculpin *Triglops nybelini* Jensen, 1944 caught by trawl in the Barents Sea in January–February 2016.

**Methods:**

Morphological preparations of the parasites were examined and photographed under a microscope at magnifications of × 100–1000 and morphometric analyses were carried out on 22 specimens using ImageJ2 software. Eight of the specimens used for the morphological comparisons were also subjected to molecular analyses by sequencing a region of the ribosomal DNA spanning partial 18S, the internal transcribed spacers 1 and 2 (ITS1 and 2), 5.8S and partial 28S and comparing this with other species through a BlastN-search in GenBank and through phylogenetic analyses.

**Results:**

The morphology of the species from *T. nybelini* was markedly different to that of any of other species of *Gyrodactylus*. It is characterized by having relatively long hamulus roots, a character that it shares with two other species described from marine sculpins (Cottidae); *G. armatus* and *G. maculosi*. It also has a narrow rectangular ventral bar membrane with a posterior notch which it shares with *G. maculosi* only. Compared with all the seven species from marine Cottidae described so far, it has the smallest opisthaptoral hard parts. A comparison of the internal transcribed spacer (ITS) rDNA sequence with available sequences in GenBank and a phylogenetic analyses also showed it to be highly divergent from other sequences. Therefore, a new species is proposed, *Gyrodactylus triglopsi* n. sp.

**Conclusion:**

Both the morphological and molecular analyses support the status of *G. triglopsi* as a new species. This is to our knowledge the first species of *Gyrodactylus* described from *Triglops nybelini* and the description extends the list of *Gyrodactylus* species found on fish in the Barents Sea to 17.

## Introduction

According to FishBase [1], the bigeye sculpin *Triglops nybelini* Jensen, 1944 is an Arctic cottid species distributed along the coasts of Greenland, at Jan Mayen and occasionally in Ungava Bay and on the Labrador coast of Canada. The specimens on which this study is based were caught in the northern Barents Sea in January–February 2016. The parasite fauna of Arctic marine fish is generally poorly known, so one of the objectives of this cruise was to collect information on the parasite faunas of fish species for which little such information was available. *Triglops nybelini* is one such species and this paper describes a monogenean of the genus *Gyrodactylus* Nordmann, 1832 found on its gills. *Gyrodactylus* is a particularly species-rich genus [2], but relatively few species have been described from Arctic waters [3], and only 16 have been reported from fish in the Barents Sea [4]. This scarcity of information probably reflects the few parasitological investigations in the area that have been performed in a way suitable for the detection of these small parasites. No *Gyrodactylus* sp. has previously been described from *T. nybelini*. The present study uses both morphological and molecular methods to describe the specimens collected from *T. nybelini* as *Gyrodactylus triglopsi* n. sp.

## Materials and Methods

Specimens of *T. nybelini* for this study were collected during a cruise of the University of Tromsø’s research vessel *Helmer Hanssen* between 25 January and 8 February 2016. Demersal trawls were made at depths ranging from 53 to 612 m and the five specimens of *T. nybelini* examined were all caught east of Svalbard at a depth of 210 m on 31 January 2016. The total length of each fish was taken, followed by a complete parasitological examination, including the examination of scrapings from the gill arches under a dissecting microscope at a magnification of × 20. Gills found infected with *Gyrodactylus* spp. were preserved, some in 10% buffered formal saline for morphological description and some in ethanol for molecular description. Parasites taken from the gills of three infected fishes preserved in ethanol were selected for morphological and molecular analyses.

### Morphological and Morphometric Analyses

Lengths and widths of whole unstained specimens and diameters of their opisthaptors were measured under magnifications of × 200–400. For measurements of opisthaptoral hard parts, the opisthaptors of 22 parasites were removed with a scalpel blade, the soft tissue was digested and the hard parts prepared for morphological analyses according to standard procedures [see, e.g., 5]. Morphological preparations were examined and photographed under a microscope (Leica DM5000) at magnifications of × 100–1000. A line drawing of the opisthaptoral hard parts was also prepared.

**Fig. 1:**
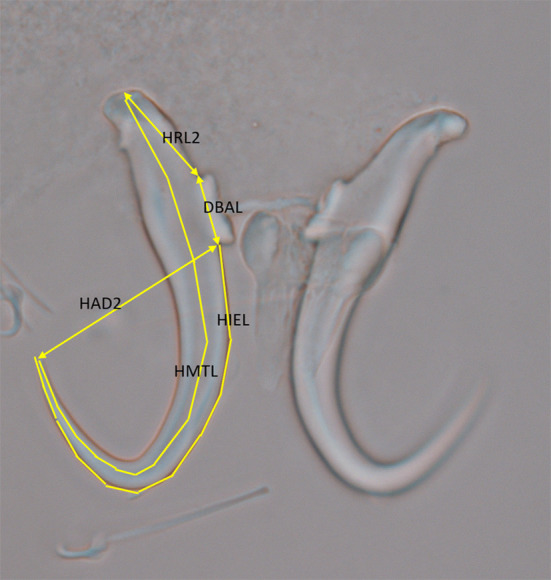
*Gyrodactylus triglopsi* n. sp*.* Image of hamuli showing the new morphometric measurements used in the current study

Measurements of the opisthaptoral hard parts were made using ImageJ2 software (version 1.52n; free download at https://imagej.net/). Several of the point to point measurements of the haptoral armature (presented in μm) were based on measurements commonly used for *Gyrodactylus* species [6]. However, it was not possible to obtain all of these measurements for *G. triglopsi* n. sp. because the new species lacks the ventral bar articulation point, a feature on which several published measurements were based. In addition, the ventral bar process length (VBPL) was omitted because *G. triglopsi* n. sp. also lacks this feature. Five new measurements were therefore added to describe the morphometry in sufficient detail (see below and Fig. 1). The new measurements were as follows: HAD2, hamulus aperture distance—from hamulus point tip to lower part of the ventral bar articulation point; HRL2, hamulus root length—from the distal edge of the hamulus to the top (beginning) of the dorsal bar attachment point; DBAL, dorsal bar attachment point length; HIEL: hamulus inner edge length—from lower part of dorsal bar attachment point, along the edge to the hamulus point tip. When taking these measurements, the same number of vectors, typically ten, was chosen for each specimen: HMTL, hamulus midline total length, from the distal edge of the hamulus along the midline of the hamulus to the point tip. As for HIEL the same number of vectors per specimen was chosen. It was not possible to obtain all morphological measurements from all specimens due to unsuitable preparations.

### Molecular Analyses

Eight of the specimens used for the morphological comparisons were also subjected to molecular analyses by sequencing the ITS rDNA region spanning the ribosomal partial 18S, the internal transcribed spacers 1 and 2 (ITS1 and 2), 5.8S and partial 28S. This fragment is the common molecular marker/barcode for species discrimination in the genus *Gyrodactylus* [see, e.g., 7, 8]. DNA was extracted from individual specimens using the DNEasyKit (Qiagen) on a QiaCube automated extraction robot in accordance with the manufacturer’s instructions. The primer pairs ITS1A and ITS2 [8] were used to amplify the specified fragment. Each PCR reaction was performed with puRe Taq Ready-to-Go PCR beads (Amersham Biosciences) in a GeneAmp PCR System 9700 (Applied Biosystems) according to the instructions from the manufacturer. The following protocol was used: 4 min at 95 °C, followed by 35 cycles of 1 min at 95 °C, 1 min at 50 °C and 2 min at 72 °C.

The PCR products were purified using a QIAquick PCR Purification Kit (Qiagen) according to the manufacturer’s recommendations. Both DNA strands were sequenced using the PCR primers on an ABI 3700XL (Applied Biosystems) using DyeET-terminator mix (GEHealthcare). Sequences were proofread in VectorNTI 11.5.4 (Invitrogen) and the sequence covering ITS1, 5.8S and ITS2 (excluding 18S and 28S) was in total 978 bp and was compared with sequences from available *Gyrodactylus* species via a GenBank BlastN search (https://www.ncbi.nlm.nih.gov/) [9]. As ITS2 alone is available from a larger number of species, a separate BlastN search was performed with this fragment (433 bp).

As mentioned by other authors [10], ITS1 is generally difficult to align reliably due to high variation in length between sequences from different species. In addition, for some species relevant to this study, only ITS2 sequences were available. Therefore, only ITS2 was used to calculate the genetic distances and for phylogenetic reconstruction. The alignment was performed using MUSCLE as implemented in MEGA X [11] and identical sequences representing the same species and sequences not covering the full ITS2 fragment were removed. There were a total of 357 positions in the final data set.

The final data set consisted of 28 nucleotide sequences from (1) available sequences from species previously found in the Barents Sea except for *G. emembranatus* Malmberg, 1970 (JF836148), which is highly divergent from the other sequences (see Table 1): *G. aeglefini* Bykhovsky and Polyansky, 1953 (JF836145), *G. arcuatus* Bykhovsky, 1933 (EF495225), *G. branchicus* Malmberg, 1964 (FJ435199), *G. groenlandicus* Levinsen, 1881 (KJ095104), *G. marinus* Bykhovsky and Polyansky, 1953 (GQ150537), *G. perlucidus* Bykhovsky & Polyansky, 1953 (FJ435202), *G. pharyngicus* Malmberg, 1964 (JF836151), *G. pterygialis* Bykhovsky and Polyansky, 1953 (AJ581657), and 2) from those with the highest BlastN hits (cover 85–100%): *G. antarcticus* Gusev, 1967 (LT719090)*, G. coriicepsi* Rokicka, Lumme & Ziętara, 2009 (FJ009451)*, G. mariannae* Winger, Hansen, Bachmann & Bakke, 2008 (DQ288255)*, G. aideni* Mullen, Cone, Easy & Burt, 2010 (HM481248)*, G. corti* Mizelle & Kritsky, 1967 (KJ095103)*, G. cyclopteri* Scyborskaya, 1948 (KP090176)*, G. flesi* Malmberg, 1957 (AY278039, AY338453)*, G. hrabei* Ergens, 1957 (DQ288253)*, G. nudifronsi* Rokicka, Lumme & Ziętara, 2009 (FJ009452)*, G. pleuronecti* Cone, 1981 (HM481247)*, G. robustus* Malmberg, 1957 (AY278040)*, G. wilkesi* Hargis and Dillon, 1968 (LT719091)*, G. adspersi* Cone and Wiles, 1983 (KJ124725)*, G. longipes* Paladini, Hansen, Fioravanti & Shinn, 2011 (GQ150536)*, Gyrodactylus* sp. DC2-01–01 (JF836153)*, Gyrodactylus* sp. JW-47 (JF836143). *Gyrodactylus bullatarudis* Turnbull, 1956 (AY692024) and *G. poeciliae* Harris & Cable, 2000 (AJ001844) were chosen as outgroup species (see Rokicka [3]). Uncorrected *p* distances between ITS2 sequences of the different species were calculated using MEGA X and pairwise deletion, removing all ambiguous positions for each sequence pair.

**Table 1:** Uncorrected *p* distances of the internal transcribed spacer (ITS2) sequence from *Gyrodactylus triglopsi* n. sp. to sequences from species of *Gyrodactylus* from the Barents Sea (top part), and to those related species with the shortest calculated *p* distance (bottom part)

*Gyrodactylus* species	Host	GenBank accession number ITS	*p*-distance to *G. triglopsi *n. sp.
Barents Sea *Gyrodactylus* spp*.* (sorted alphabetically)			
*Gyrodactylus aeglefini*	*Melanogrammus aeglefinus*	JF836145	0.198
*Gyrodactylus anarhichatis*	*Anarhichas lupus*	NA	NA
*Gyrodactylus arcuatus*	*Salmo salar*	EF495225	0.245
*Gyrodactylus branchicus*	*Gasterosteus aculeatus*	FJ435199	0.189
*Gyrodactylus callariatis*	*Gadus morhua*	NA	NA
*Gyrodactylus cryptarum*	*Gadus morhua*	NA	NA
*Gyrodactylus dogieli*	*Limanda limanda*	NA	NA
*Gyrodactylus emembranatus*	*Gadus morhua*	JF836148	0.400
*Gyrodactylus errabundus*	*Zoarces viviparus*	NA	NA
*Gyrodactylus gerdi*	*Eleginius navaga*	NA	NA
*Gyrodactylus groenlandicus*	*Myoxocephalus scorpius*	KJ095104	0.077
*Gyrodactylus marinus*	*Gadus morhua*	GQ150537	0.195
*Gyrodactylus microanchoratus*	*Anarhichas lupus*	NA	NA
*Gyrodactylus perlucidus*	*Zoarces viviparus*	FJ435202	0.102
*Gyrodactylus pharyngicus*	*Gadus morhua,*	JF836151	0.178
*Gyrodactylus pterygialis*	*Gadus morhua*	AJ581657	0.190
With shortest *p*-distance (sorted by distance)			
*Gyrodactylus aideni*	*Pleuronectes americanus*	HM481248	0.081
*Gyrodactylus adspersi*	*Anarrhichthys ocellatus*	KJ124725	0.082
*Gyrodactylus pleuronecti*	*Pleuronectes americanus*	HM481247	0.084
*Gyrodactylus antarcticus*	*Trematomus newnesi*	LT719090	0.090
*Gyrodactylus wilkesi*	*Trematomus bernacchi*	LT719091	0.091
*Gyrodactylus coriicepsi*	*Notothenia coriiceps*	FJ009451	0.097
*Gyrodactylus corti*	*Anarrhichthys ocellatus*	KJ095103	0.102
*Gyrodactylus mariannae*	*Cottus poecilopus*	DQ288255	0.104
*Gyrodactylus hrabei*	*Cottus poecilopus*	DQ288253	0.109
*Gyrodactylus cyclopteri*	*Cyclopterus lumpus*	KP090176	0.113
*Gyrodactylus nudifronsi*	*Lepidonotothen nudifrons*	FJ009452	0.120
*Gyrodactylus flesi*	*Platichthys flesus*	AY278039	0.127
*Gyrodactylus robustus*	*Platichthys flesus*	AY278040	0.127
*Gyrodactylus flesi*	*Pleuronectes platessa*	AY338453	0.127
*Gyrodactylus longipes*	*Sparus aurata*	GQ150536	0.130
*Gyrodactylus* sp._JW-47	*Cottus asper*	JF836143	0.158
*Gyrodactylus* sp._DC2-01–01	*Microgadus tomcod*	JF836153	0.190

All accession numbers listed in the table, except for the one from *G. emembranatus*, are included in the phylogenetic analyses

Phylogenetic relationships were inferred by neighbor-joining and maximum likelihood (ML) with MEGA X [11]. The neighbor-joining analysis was performed using the maximum composite likelihood of calculating evolutionary distances and with gamma-distributed rates among sites. Nodal support was estimated by bootstrapping (*n* = 1000). The best model of evolution was calculated in MEGA X [11] and selected based on the Akaike information criterion; GTR + G was chosen for each partition. For ML, an initial tree was estimated using the setting NJ/BioNJ followed by a heuristic search performed implementing the estimated model parameters using nearest-neighbor interchange (NNI) branch swapping. All sites were used in the analyses. Nodal support was estimated by ML bootstrapping (*n* = 1000).

## Results

The five sculpins caught measured from 10 to 13 cm in total length. Three were females and two were males. Gyrodactylids were present on the gill filaments of all five fish. Gills from three fish were examined in detail and the intensity of infection varied from 15 to > 50 parasites per fish. The parasites were found on both the gill arches and filaments. Of the 18 specimens subjected to digestion of the opisthaptoral hard parts, 5 preparations were found unsuitable for further analyses. Morphological examination of the remaining 13 specimens revealed that they represented a single morphological species.

### Taxonomic Summary

*Type host*: bigeye sculpin *Triglops nybelini* Jensen, 1944.

*Site of infection*: gill filaments and gill arches.

*Type locality*: northern Barents Sea east of Svalbard, 77°58ˊN X 30°36ˊE, depth 210 m.

*Type material:* one holotype (NHMO C 7037) and six paratypes (NHMO C 7038–7043) are deposited in the Natural History Museum, Oslo, Norway.

*Etymology:* named after its type host *Triglops nybelini* Jensen, 1944.

### Description

All measurements are presented in µm below as the mean ± standard deviation (SD), followed, in parentheses, by the range and the number of specimens measured for that particular feature. Measurements are given to the nearest micrometer except for some measurements of marginal characters. The description is based on whole body and opisthaptoral measurements of 17 specimens and the opisthaptoral hard parts of 22 specimens.

**Fig. 2:**
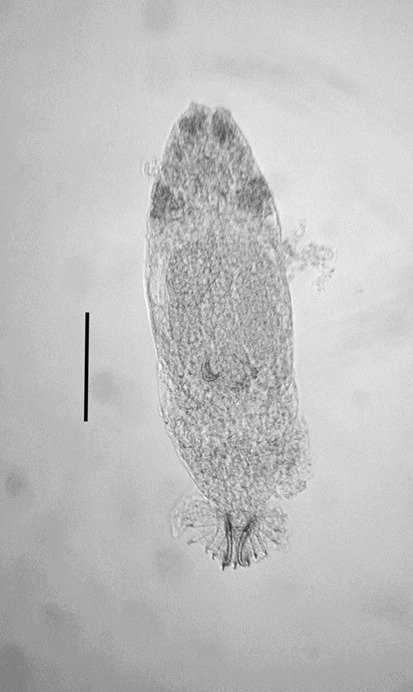
*Gyrodactylus triglopsi* n. sp. Whole unstained specimen fixed in 10% formalin. Scale bar = 100 μm

Total body length 415 ± 62.0 (275–550) (*n* = 17), width at uterus 105 ± 15.3 (90–140) (Fig. 2). Opisthaptor diameter 51.6 ± 5.03 (40–60) (*n* = 17) (Fig. 2).

**Fig. 3:**
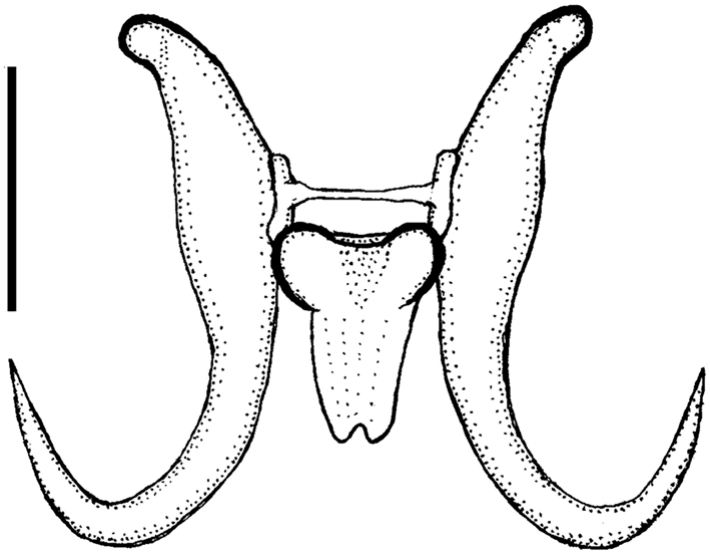
*Gyrodactylus triglopsi* n. sp. Line drawing of the hamuli and ventral bar. Scale bar = 20 μm

**Fig. 4:**
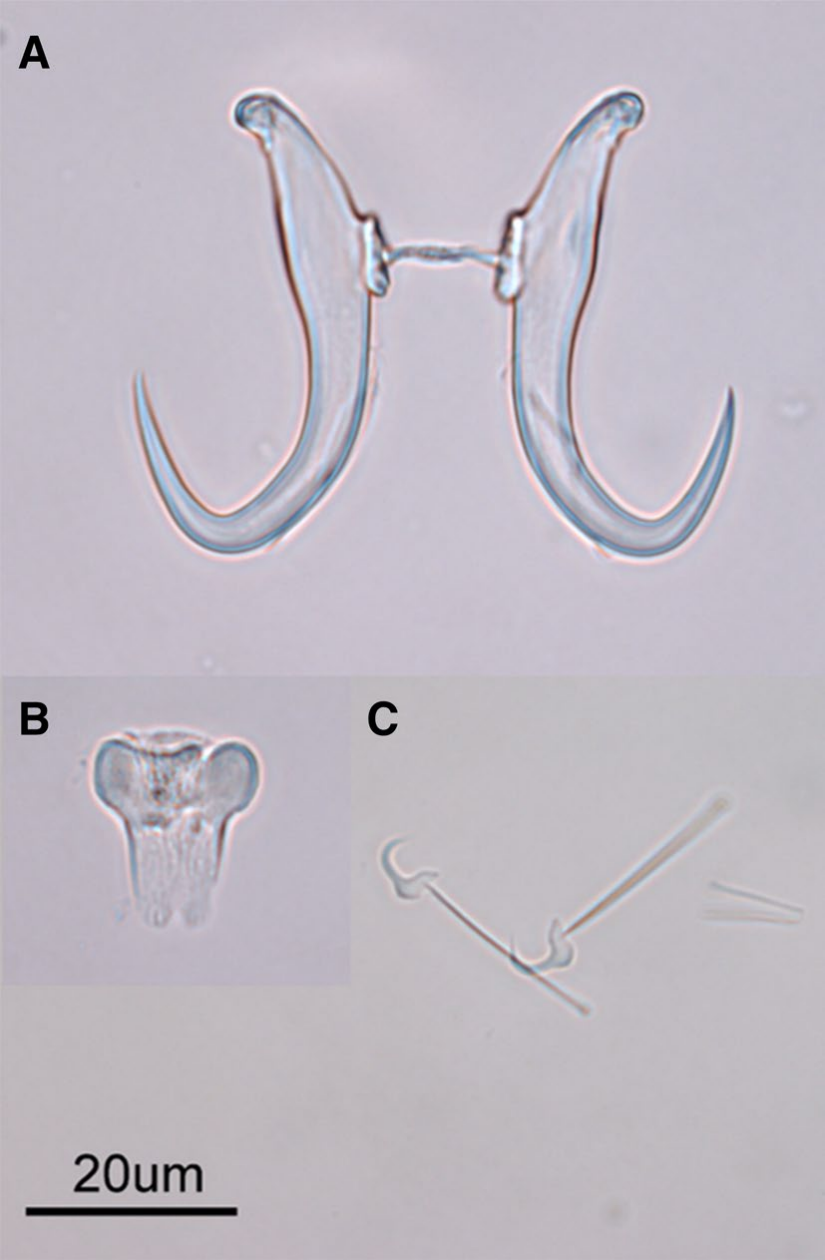
*Gyrodactylus triglopsi* n. sp. Photographs of the different components of opisthaptoral hardparts. **a** Hamuli, **b** ventral bar, and **c** marginal hooks. Scale bar = 20 μm

Hamulus (Figs. 1, 3 and 4a), with relatively long root, wide around the dorsal bar attachment point and lacking the ventral bar articulation point. Total length (HTL) 43 ± 1.8 (38–46) (*n* = 22), root length (HRL) 19 ± 1.3 (16–20) (*n* = 22), root length 2 (HRL2) 15 ± 1.2 (12–17) (*n* = 22), aperture distance (HAD2) 24 ± 1.2 (21–26) (*n* = 22), dorsal bar attachment point length (DBAL) 7.5 ± 0.8 (6–9) (*n* = 22), inner edge length (HIEL) 51 ± 1.4 (47–52) (*n* = 22), midline total length (HMTL) 66 ± 2.1 (61–69) (*n* = 22).

Ventral bar (Figs. 3 and 4b), narrow with rectangular membrane and posterior notch. Ventral bar total width (VBTW) 16 ± 0.9 (14–18) (*n* = 13), ventral bar total length (VBTL) 18 ± 1.0 (16–19) (*n* = 13), ventral bar process-to-mid length (VBPML) 1 ± 0.3 (0.7–1.6) (*n* = 11), ventral bar median length (VBML) 7 ± 1.6 (5–9) (*n* = 12), ventral bar membrane length (VBMBL) 10 ± 1.0 (8–12) (*n* = 12).

**Fig. 5:**
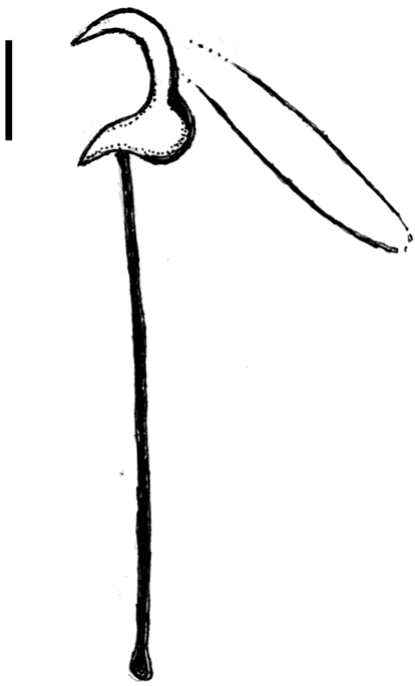
*Gyrodactylus triglopsi* n. sp. Line drawing of marginal hook. Scale bar = 5 μm

Marginal hooks (Figs. 4c and 5), total length (MHTL) 25 ± 0.4 (24–26) (*n* = 16), shaft length (MHSHL) 20 ± 0.4 (19–20) (*n* = 16), sickle length (MHSL) 5.8 ± 0.2 (5.5–6.1) (*n* = 16), sickle proximal width (MHSPW) 4.2 ± 0.2 (3.8–4.6) (*n* = 16), sickle distal width (MHSDW) 4 ± 0.2 (3.6–4.4) (*n* = 16), toe length (MHSTL) 1.6 ± 0.4 (1.3–2.9)(n = 16), aperture distance (MHAD) 4.6 ± 0.3 (4.3–5.2) (*n* = 16), instep arch/height (MHIH) 0.5 ± 0.1 (0.4 –0.8) (*n* = 14).

### Molecular Characterization

A non-variable 1056 bp PCR product covering partial 18S (28 bp), ITS1 (388 bp), 5.8S (157 bp), ITS2 (433 bp), and partial 28S (50 bp) was recovered from eight specimens and submitted to GenBank under accession number KX443484.

The BlastN search [9] in June 2019 using the 978 bp sequence covering ITS1, 5.8S and ITS2 (excluding 18S and 28S) revealed no identical or close hits (max. identity ≈ 92%). The BlastN search of the ITS2 fragment alone gave the same result.

Sequences of the internal transcribed spacer were available for 9 of the 16 species reported from Barents Sea fish [4] and for 3 of these (*G. aeglefini*, *G. emembranatus*, and *G. pharyngicus*), only ITS2 sequences were available. Based on the calculations of uncorrected p distances, *G. groenlandicus* was the most closely related species, followed by *G. aideni*, *G. adspersi, G. pleuronecti, G. wilkesi* and *G. antarcticus*.

## Discussion

**Fig. 6:**
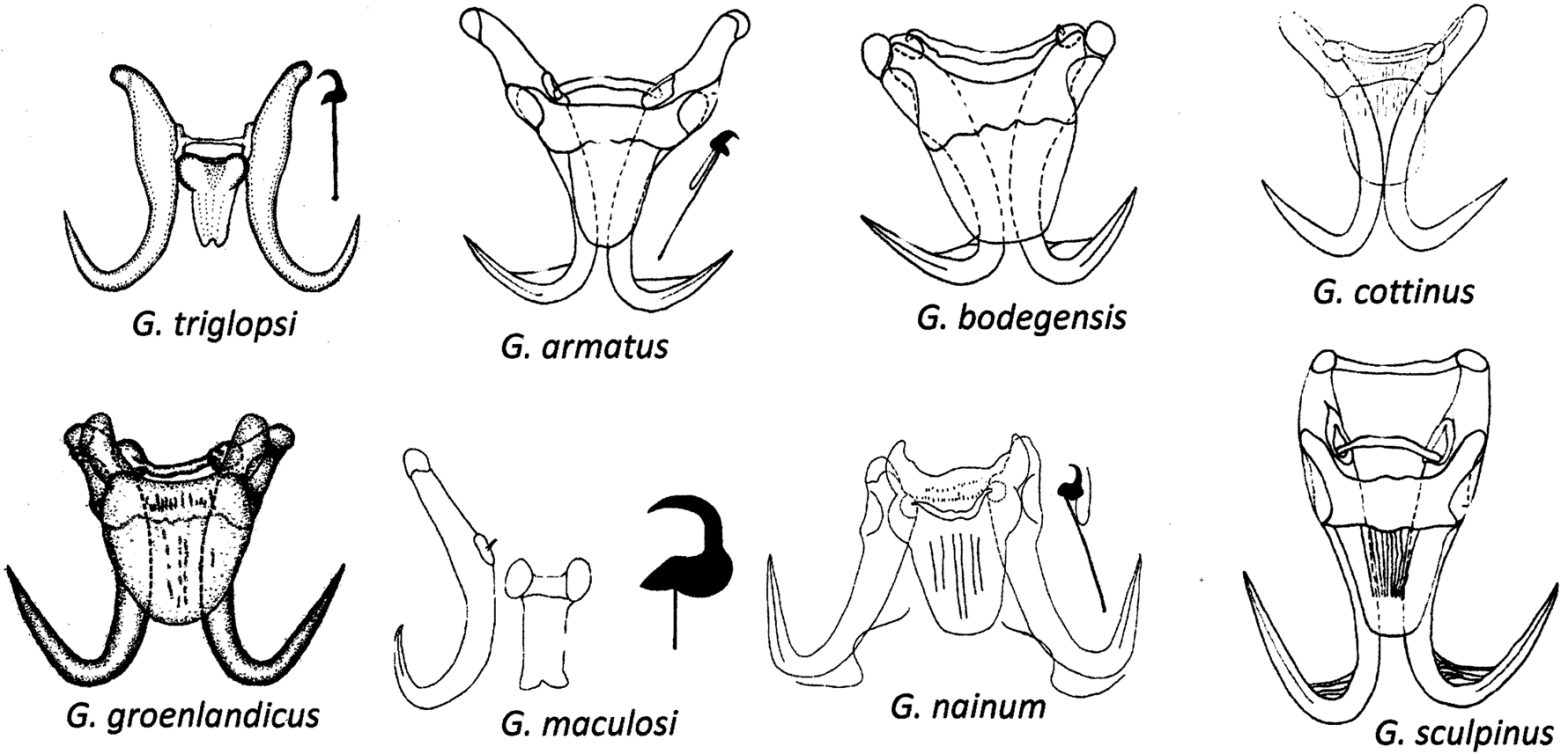
Opisthaptoral hard parts of *Gyrodactylus* spp. described from marine fishes of the family Cottidae [modified from 15–20]. Drawn approximately to scale

The parasite fauna of Arctic marine fish is generally poorly known, and prior to this study only 16 species of *Gyrodactylus* had been reported from the Barents Sea [4]. Seven species of *Gyrodactylus* have been reported from marine sculpins of the family Cottidae: *G. armatus* Crane & Mizelle, 1967, *G. bodegensis* Mizelle & Kritsky, 1967, *G. cottinus* Zhukov, 1960, *G. groenlandicus* Levinsen, 1881*, G. maculosi* Cone & Roth, 1993, *G. nainum* Hanek & Threlfall, 1970, and *G. sculpinus* Crane & Mizelle, 1967. Four of these species have relatively short hamuli roots, constituting less than 30% of hamuli total lengths, the exceptions being *G. armatus, G. maculosi* and *G. triglopsi* n. sp. In *G. maculosi* and *G. triglopsi* n. sp. the hamulus root make up > 40% of the total hamulus length, while *G. armatus* is intermediate (Fig. 6). All these species have wide ventral bars (VB) harboring ventral bar processes, with the exception of *G. maculosi* and *G. triglopsi* n. sp. These two species share long hamuli roots and narrow ventral bars without processes. They also share a narrow rectangular VB membrane with a posterior notch, and similar marginal hooks. *Gyrodactylus triglopsi* n. sp., however, is readily distinguished from *G. maculosi* by its shorter hamuli and narrower VB. *Gyrodactylus triglopsi* n. sp. has the smallest opisthaptoral hard parts of all the seven species from marine Cottidae and is the first *Gyrodactylus* species described from a fish of the genus *Triglops.*

Among the Arctic and northern marine species from non-cottid hosts, *G*. *triglopsi* n. sp. most closely resemble some members of the *Gyrodactylus marinus* group of Malmberg [12]: *G. aeglefini* Bykhovsky and Polyansky, 1953 and *G. cryptarum* Malmberg, 1970 described from marine gadid hosts in high northern latitudes. While showing similarities in VB structure, *G. triglopsi* n. sp. is readily distinguished by the short VB lacking VB processes and smaller hamuli.

**Fig. 7:**
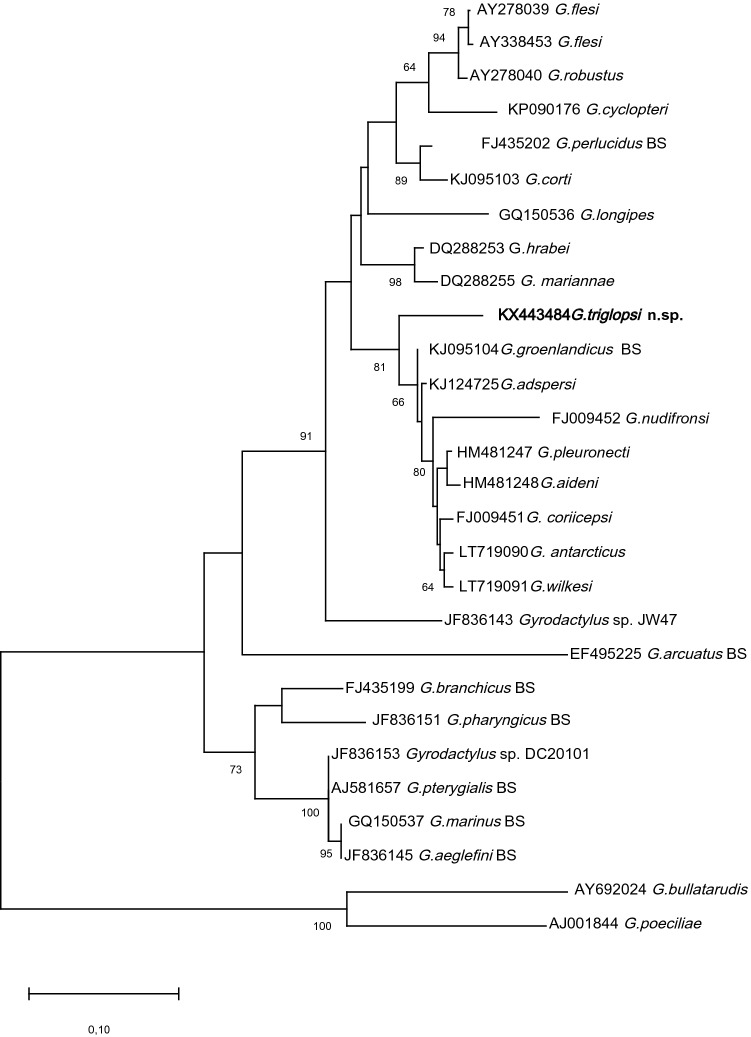
Phylogenetic tree based on maximum likelihood analysis of the ribosomal internal transcribed spacer 2 (ITS2) sequences. Nodal support was estimated by bootstrapping (*n* = 1000). Only bootstrap values above 50 are shown

Based on the comparison of genetic distances, the most closely related species to *G. triglopsi* n. sp. is *G. groenlandicus*, a sculpin parasite found in the Barents Sea. However, the distance between *G. triglopsi* n. sp. and *G. groenlandicus* far exceeds the 1% difference that is suggested for separate species status in the genus [7]. None of the analyses grouped *G. triglopsi* n. sp. with high support with any other species, which might be expected given the genetic difference to other species. There is some support for a larger grouping where *G. triglopsi* n. sp. is basal to a group with other marine species (*G. groenlandicus, G. adspersi*, *G. nudifronsi*, *G. pleuronecti*, *G. aideni*, *G. coriicepsi*, *G. antarcticus,* and *G. wilkesi*) and two species (*G. mariannae* and *G. hrabei)* infecting freshwater cottids (*Cottus* spp.) in both analyses (only ML-analyses shown, Fig. 7). The main groupings recovered in our phylogenetic analyses correspond well with earlier analyses [13, 14] with minor differences, mostly due to the fact that not all species were available in earlier studies. The overall phylogeny is thus not discussed further here. It is worth noting, however, that the sequence used for *G. corti* (KJ124725) in Heglasova et al. [13] was later changed in NCBI GenBank and now belongs to *G. adspersi*. The correct accession numbers for *G. corti* and *G. adspersi* are used here and, as in King et al. [14] *G. corti* and *G. perlucidus* form a well-supported group, while *G. adspersi* is most closely related to *G. groenlandicus*. The phylogenetic analyses also clearly show that the species from the Barents Sea (labelled BS in Fig. 7) are found in different phylogenetic groups.

The host, *Triglops nybelini*, appears to be more common along the coast of Greenland and Labrador than in the Barents Sea [1]. In our study, we caught only five specimens of *T. nybelini* in the course of five cruises in the Barents Sea from 2016 to 2018. Its congener *T. murrayi* Günther, 1888¸ however, was much more common in our catches and we examined 67 specimens of this species. Other cottid species examined were *Artediellus atlanticus* Jordan & Evermann, 1898 (32 specimens) and *Icelus bicornis* (Reinhardt, 1840) (5 specimens). None of these had a gyrodactylid infection. *Gyrodactylus triglopsi* n. sp.*,* like many species of *Gyrodactylus*, may thus be host specific [21]*.*

In conclusion, both the molecular and morphological analyses presented herein support the status of *G. triglopsi* as a new species.
